# Evidence for the effects on the wildlife gut microbiome by grazing: The potential gut microbiota transmission between Yunnan snub-nosed monkeys (*Rhinopithecus bieti*) and sympatric livestock

**DOI:** 10.1016/j.isci.2025.114147

**Published:** 2025-11-19

**Authors:** Wancai Xia, Chenyi Gao, Xinyuan Cui, Hong Li, Xueyu Wang, Fan Wang, Lifeng Zhu, Dayong Li

**Affiliations:** 1 Key Laboratory of Southwest China Wildlife Resources Conservation (Ministry of Education), China West Normal University, Nanchong, Sichuan Province 637009, China; 2 Key Laboratory of Conservation Biology of Rhinopithecus roxellana at China West Normal University of Sichuan Province, Nanchong, Sichuan Province 637009, China; 3 School of Medicine, Nanjing University of Chinese Medicine, Nanjing, Jiangsu Province 210023, China

**Keywords:** Microbiology, Cell biology

## Abstract

Grazing can impact wildlife by resource competition, habitat degradation, and pathogen transmission. The Yunnan snub-nosed monkeys (*Rhinopithecus bieti*) are endemic and endangered primates, facing the negative effects of grazing. In the study, we conducted 16S rRNA sequencing to investigate the gut microbiota of Yunnan snub-nosed monkeys and sympatric livestock. Our results revealed that cattle exhibited relatively higher microbial similarity with monkeys compared to pigs. The SourceTracker analysis further indicated a potential cattle-origin gut microbiome in monkeys (mean ± standard deviation (SD): 11.24% ± 0.43%), while no pig-derived microbiome was detected. We speculated that shared environment and partial dietary similarities may drive the microbial similarity and transmission. Furthermore, our findings suggested potential microbial transmission between the gut microbiome of livestock and the environment, revealing probable environmental influence caused by grazing. Overall, our study showed the impacts of grazing on the wildlife microbiome and the environment and provided insights for conservation management.

## Highlights

• The Yunnan snub-nosed monkey’s gut microbiota was more similar to cattle than to pigs
• The Yunnan snub-nosed monkey’s gut microbiota harbored cattle-derived microbiota
• Shared environments and diets were potential drivers of microbial transmission
• Grazing may impact the environment through microbial transmission

## Graphical abstract

**Figure:**
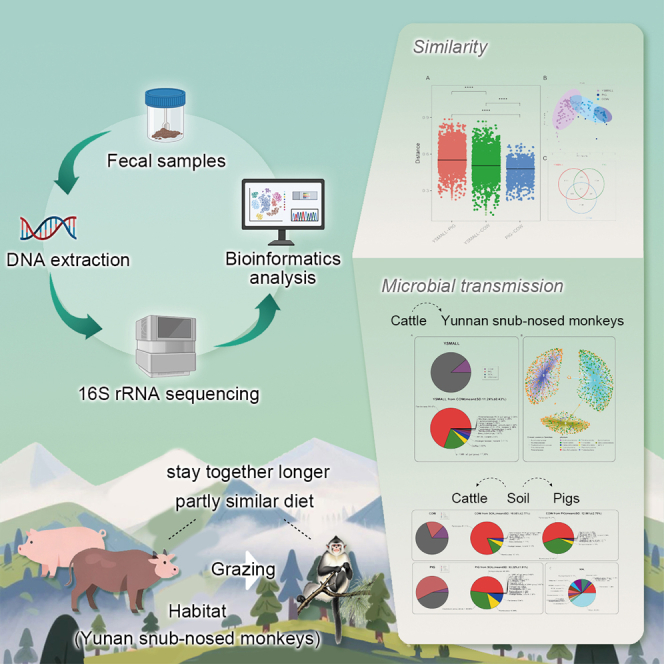

## Introduction

Dietary competition, the spread of diseases, and predation are considered primary sources of fueling conflicts between wild animals and livestock.^1^ Studies in Indian wildlife sanctuaries have revealed dietary niche overlap between wild and domestic herbivore species.^2^ Food resources for Brandt’s voles (*Lasiopodomys brandtii*) can be modified by prolonged sheep grazing, potentially inducing dietary shifts that may lead to gut microbiome alterations.^3^ Furthermore, the One Health concept underscores the interconnectedness of human, animal, and environmental health, advocating for a holistic approach to address shared challenges. It highlights the risks of transmission of pathogens and antibiotic resistance genes at the human-wildlife-livestock interface.^4^^,^^5^ Given these risks, increasing research has focused on exploring microbial pathogens and antibiotic resistance genes between wildlife and sympatric livestock. Both wildlife and livestock can serve as potential transmission hosts for pathogenic microorganisms and antibiotic resistance genes. It has been shown that regions with frequent wildlife-livestock interactions exhibit higher prevalence of pathogenic bacteria (e.g., *Brucella* spp.*, Leptospira* spp.*, Salmonella* spp.*, and Campylobacter* spp.) compared to areas with minimal contact.^6^^,^^7^ Additionally, antibiotic residues excreted in livestock feces can contaminate the ecosystems,^8^ potentially disseminating antibiotic resistance genes to wildlife populations through environmental vectors, including soil and water runoff.^9^^,^^10^ Grounded in the “One Health” framework, this study investigated changes in gut microbial communities between sympatric wildlife and livestock. It further examined how grazing influences the wildlife gut microbiome and facilitates potential pathogen transmission at the wildlife-livestock interface, which provides novel insights for promoting sustainable livestock management and informing effective wildlife conservation strategies.

The gut microbiome is crucial for the health, environmental adaptation, and survival of wildlife, which influences nutrient metabolism, immune function, and host evolution.^11^^,^^12^^,^^13^^,^^14^^,^^15^ The Yunnan snub-nosed monkeys (*Rhinopithecus bieti*) are listed as critically endangered on the IUCN Red List,^16^ facing significant threats due to habitat fragmentation, climate change, and human activities such as hunting pressure.^17^^,^^18^^,^^19^ As a non-human primate (NHP) phylogenetically close to humans,^20^ the Yunnan snub-nosed monkeys serve as an ideal model for studying gut microbiome dynamics. Investigating gut microbial changes in Yunnan snub-nosed monkeys can provide a theoretical foundation for wildlife conservation and management and deepen our insight into the adaptive evolution of the human gut microbiome.

Host phylogeny, environment, diet, and social behavior are recognized as the main factors shaping gut microbial communities.^21^^,^^22^^,^^23^^,^^24^^,^^25^ Significant divergences in gut microbiome structure and function have been observed between captive and wild specimens of Rhinopithecus species (*Rhinopithecus roxellana* and *Rhinopithecus brelichi*), reflecting dietary adaptation.^26^^,^^27^ Furthermore, interspecific comparisons among Rhinopithecus species have demonstrated differences in gut microbiomes and underscored the influence of phylogeny, diet, and habitat on microbial composition and function.^28^^,^^29^ Notably, anthropogenic food provisioning has been shown to lead to convergence in gut microbiome functions and antibiotic resistance gene profiles.^30^ Most current research on the snub-nosed monkey gut microbiome has focused on comparative analysis across different environments or species and the factors influencing these differences. However, the gut microbiome composition and interspecies microbial associations between Yunnan snub-nosed monkeys and domestic livestock remain poorly investigated.

The Yunnan snub-nosed monkey can be classified as herbivores; their possesses a specialized stomach structure^31^ that enables it to consume a diverse array of foods abundant in structural polysaccharides, such as flowers, fruits, leaves, and seeds. Our previous research on the dietary patterns of this primate group revealed their consumption of 188 distinct plant parts from 105 plant species across 42 families.^32^ This included 34 tree species, 27 shrub species, 12 vine species, 16 herb species, 3 parasitic plant species, 4 lichen species, and 9 fungi species. The primary components of the Yunnan snub-nosed monkeys' yearly diet were Rosaceae and Gramineae, except lichen. In the context of domestic animals, while pigs and cattle encounter challenges in accessing food resources located in tall tree canopies akin to those utilized by Yunnan snub-nosed monkeys, they can still consume low shrubs, herbs, and ripe fallen fruits. The Yunnan snub-nosed monkeys observed in this study are semi-habituated, residing within the Baimaxueshan Nature Reserve, which. In the Xiangguqing region, domestic pigs are typically released in the early morning and herded back to their enclosures in the evening, with their movement and time frame strictly regulated by humans. Conversely, domestic cattle enjoy year-round freedom to graze. This disparity in grazing behavior results in domestic cattle having more opportunities to forage on understory shrubs and herbs, thereby heightening the likelihood of overlapping with the feeding niche of monkeys. semi-habituated. Prior studies have highlighted concerns regarding the dissemination of antibiotic resistance genes (ARGs) and the transmission potential of pathogens at the interface between wildlife and domesticated animals.^9^^,^^33^ Building upon this context, our study pursued three primary objectives: (1) to compare the gut microbial communities of Yunnan snub-nosed monkeys and livestock; (2) to determine whether microbial similarity is greater between Yunnan snub-nosed monkeys and cattle than between monkeys and pigs, based on shared environmental exposure and partial dietary overlap; and (3) to evaluate how free-range livestock grazing may influence wildlife gut microbiome. Gaining a clearer understanding of microbial shifts across wildlife-livestock boundaries is crucial for informing conservation strategies and mitigating risks of interspecies disease transmission.

## Results

### The gut microbiome composition of Yunnan snub-nosed monkeys and livestock

**Figure 1:**
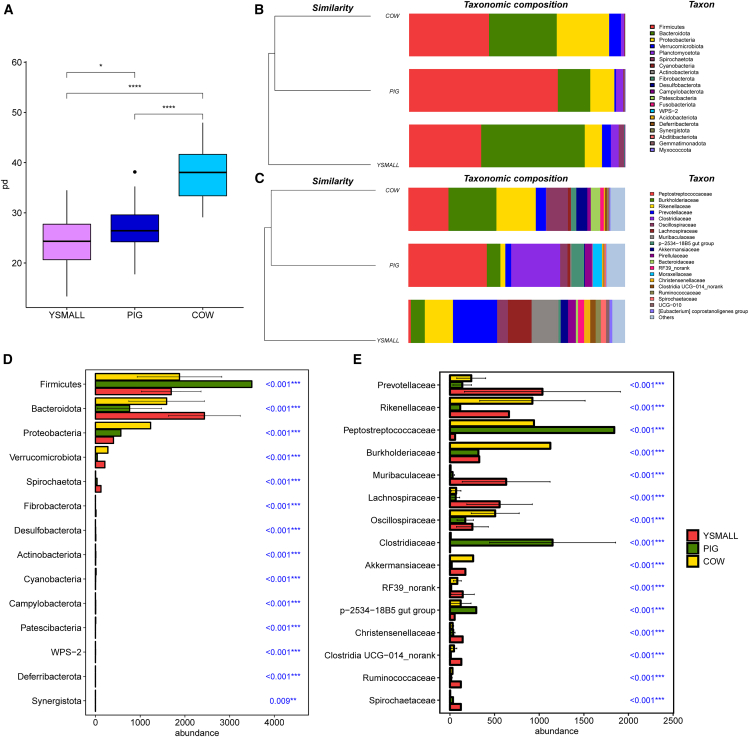
Gut microbial composition in Yunnan snub-nosed monkeys (YSMALL), pigs (PIG), and cattle (COW). *n* = 106 for YSMALL, *n* = 30 for PIG, *n* = 29 for COW. Data are represented as mean ± SEM. ∗*p* < 0.05, ∗∗∗*p* < 0 0.001, ∗∗∗∗*p* < 0.0001 (A)Alpha diversity analysis of gut microbiome based on the Phylogenetic Diversity (PD). (B and C) Relative abundances of dominant bacterial (B) phyla and (C) families in the gut microbiome of Yunnan snub-nosed monkeys, pigs, and cattle. (D and E) Taxa with significant differences in the gut microbiome at the (D) phylum and (E) family level in Yunnan snub-nosed monkeys, pigs, and cattle.

Analysis of alpha diversity (Phylogenetic Diversity, Chao1, Shannon, and Simpson indices) showed that the gut microbiome diversity was highest in cattle, followed by pigs, with Yunnan snub-nosed monkeys showing the lowest diversity (Figures 1A and S1A–S1C). Furthermore, the analysis of gut microbial composition revealed that at the phylum level, the gut microbiome of the three species was primarily composed of Firmicutes, Bacteroidota, and Proteobacteria, but their relative abundances exhibited distinct variations across species (Figures 1B, 1D, S2, and S3; Table S1). Specifically, pigs displayed the highest abundance of Firmicutes, whereas Yunnan snub-nosed monkeys were enriched in both Firmicutes and Bacteroidota. In contrast, cattle maintained high relative abundances of all three phyla. Taxonomic analysis at the family level revealed distinct compositional differences: while Peptostreptococcaceae and Clostridiaceae were predominant in pigs, cattle displayed higher proportions of Peptostreptococcaceae along with Burkholderiaceae and Rikenellaceae. The Yunnan snub-nosed monkeys were found enriched in Muribaculaceae, Prevotellaceae, Rikenellaceae, and Lachnospiraceae. Notably, the distribution of these microbial taxa was significantly different across species (Figures 1C, 1E, S2, and S3; Table S2).

### The gut microbiome exhibited greater similarity in Yunnan snub-nosed monkeys and cattle

**Figure 2:**
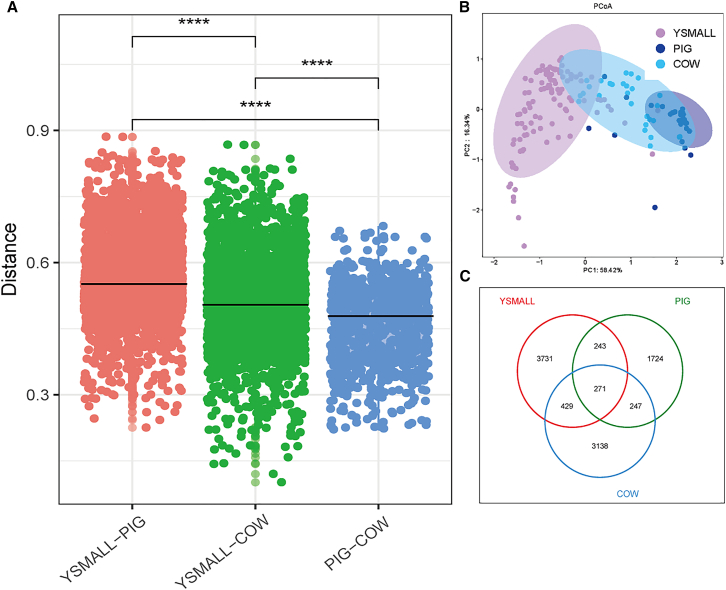
Similarity of gut microbiome between Yunnan snub-nosed monkeys and cattle. *n* = 106 for YSMALL, *n* = 30 for PIG, *n* = 29 for COW. ∗∗∗∗*p* < 0.0001 (A) Comparative analysis of differences between groups based on weighted UniFrac distances. (B) Principal coordinate analysis (PCoA) based on weighted UniFrac distances, illustrating gut microbiome composition across different groups. (C) Venn diagram illustrating the number of amplicon sequence variant (ASVs) shared between the gut microbiome of Yunnan snub-nosed monkeys, pigs, and cattle.

**Figure 3:**
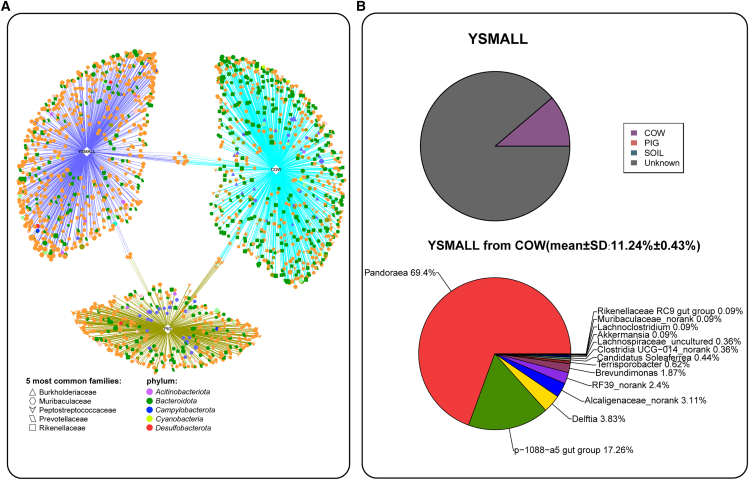
The potential microbial transmission between Yunnan snub-nosed monkeys and cattle. *n* = 106 for YSMALL, *n* = 29 for COW. Data are represented as mean ± SEM (A) Network analysis shows the same indicator species that may be present between groups. Each node represents an amplicon sequence variant (ASV). Node size corresponds to relative abundance, and node color indicates phylum-level taxonomy. line color corresponds to the group color, line thickness denotes strength, and line length represents the degree of correlation. (B) SourceTracker analysis of gut microbiome in Yunnan snub-nosed monkeys and the genus-level categorization of potential contribution source microbiome.

Comparative analysis of intergroup dissimilarities based on weighted UniFrac distances confirmed that the gut microbial divergence between Yunnan snub-nosed monkeys and cattle was significantly smaller than that between Yunnan snub-nosed monkeys and pigs (Figure 2A). Furthermore, principal coordinates analysis (PCoA) of weighted UniFrac distance revealed distinct clustering patterns in the gut microbiome of pigs and Yunnan snub-nosed monkeys. In contrast, the microbiome distribution of cattle was more similar to Yunnan snub-nosed monkeys (Figure 2B). From the perspective of gut microbial composition, a higher proportion of Bacteroidota and reduced Firmicutes levels were observed in the gut microbiomes of cattle and Yunnan snub-nosed monkeys relative to those of pigs (Figure 1B). At the family level, both species showed higher abundances of Rikenellaceae and Prevotellaceae, but a relatively lower abundance of Peptostreptococcaceae (Figure 1C). Subsequent analysis of shared amplicon sequence variant (ASVs) demonstrated greater microbial overlap between Yunnan snub-nosed monkeys and cattle (700 ASVs) compared to Yunnan snub-nosed monkeys and pigs (514 ASVs) (Figure 2C). In addition, the indicator analysis identified richer indicator species in the Yunnan snub-nosed monkeys-cattle group compared to the Yunnan snub-nosed monkeys-pigs group (Figure 3A). These findings further prove higher gut microbiome similarity between Yunnan snub-nosed monkeys and cattle between Yunnan snub-nosed monkeys and pigs.

### The microbiome may transfer through interspecies interactions and the environment

**Figure 4:**
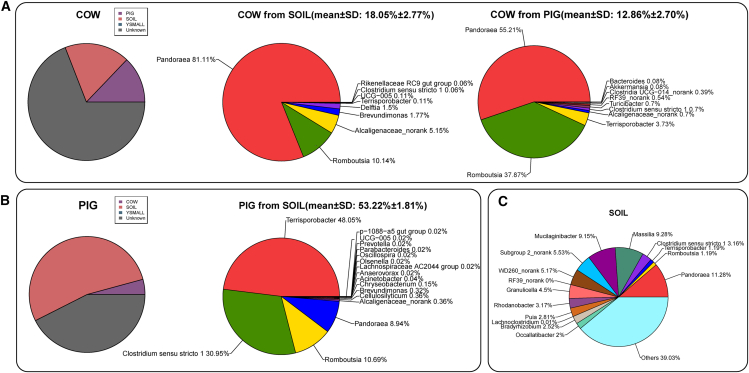
The potential microbial transmission via the environment. *n* = 30 for PIG, *n* = 29 for COW, *n* = 10 for SOIL. Data are represented as mean ± SEM (A) SourceTracker analysis of gut microbiome in cattle and the genus-level categorization of potential contribution source microbiome. (B) SourceTracker analysis of gut microbiome in pigs and the genus-level categorization of potential contribution source microbiome. (C) Classification of microorganisms in soil at the genus level.

The SourceTracker analysis revealed that the gut microbiome of cattle has a potential contribution to Yunnan snub-nosed monkeys (mean ± standard deviation (SD): 11.24% ± 0.43%), with *Pandoraea* accounting for 69.4%. In contrast, no potential pig-derived microbiome was detected in Yunnan snub-nosed monkeys (Figure 3B). Indicator species analysis further identified indicator species shared between Yunnan snub-nosed monkeys and cattle (Figure 4B). These results suggested that microbial transmission may occur between cattle and Yunnan snub-nose monkeys. In addition, we also observed that the 12.86% putative contribution of the pig gut microbiome to the cattle gut microbiome (Figure 4A).

Beyond inter-host microbial transmission, the SourceTracker analysis also demonstrated that the soil microorganisms may serve as a potential source for the livestock gut microbiome. We found that the soil-derived microbiome may contribute to the cattle gut microbiome (mean ± SD: 18.05% ± 2.77%), of which 81.11% were classified as *Pandoraea* (Figure 4A). In contrast, the putative contribution of soil microbiome showed significantly higher to pigs (mean ± SD: 53.22% ± 1.81%), primarily consisting of *Terrisporobacter*, *Clostridium_sensu_stricto_1*, and *Romboutsia* (Figure 4B).

## Discussion

### Grazing may pose a risk of contaminating the environment and influencing sympatric hosts

Our study revealed that the gut microbiome of pigs was enriched in *Clostridium_sensu_stricto_1*, *Terrisporobacter*, and *Romboutsia* (Figure S1), consistent with previous findings.^34^ Notably, a recent study investigated how the pig gut microbiome affects the environment and occupationally exposed humans. The results revealed that occupationally exposed farmers and the surroundings of pig farms contained high proportions of *Clostridium_sensu_stricto_1 and Terrisporobacter,* which were also found to be present in high abundance in the gut microbiome of pigs.^35^ These bacterial genera were recognized as commensal bacteria in humans and environmental reservoirs and identified as potential carriers of antibiotic resistance genes (ARGs) in pig feces.^34^ Moreover, they have been strongly associated with human pathologies, including colitis, atherosclerosis, neuropsychiatric disorders, type 1 diabetes, and so on.^36^^,^^37^^,^^38^^,^^39^ These findings suggested the potential influence of grazing on contaminating the environment, spreading the pathogen and ARGs, and affecting human health. Similarly, our analysis confirmed that the intestinal microbiome of pigs plays a role in shaping environmental and host-related dynamics. Our analysis revealed the soil microorganisms contained *Clostridium_sensu_stricto_1* (3.16%), *Romboutsia* (1.19%), and *Terrisporobacter* (1.19%) (Figure 4C), which may reflect environmental contamination by pig-derived gut microbiome. In addition, in the cattle gut microbiome that potentially originates from soil and pigs, high proportions of *Terrisporobacter* and *Romboutsia* were detected, suggesting potential microbial transmission at the environment-host interface and between different host species. Our findings indicated that grazing may lead to the contamination of local ecosystems, potentially affecting sympatric host species through direct contact or environmental transmission pathways. Furthermore, it may facilitate the environmental dissemination of pathogenic bacteria, posing significant risks to environmental and human health.

### Environment and diet may drive the higher similarity in gut microbial composition between Yunnan snub-nosed monkeys and cattle

**Figure 5:**
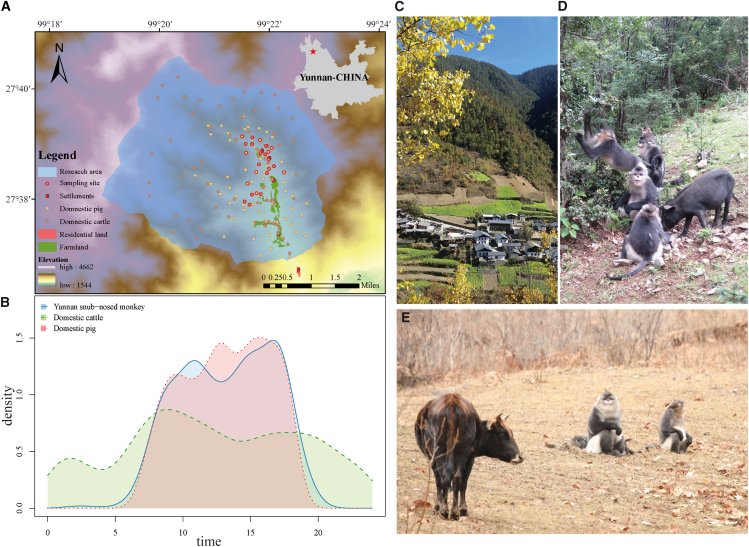
Study area and livestock distribution (A) Geographical overview of the study area, showing the spatial distribution of the residential regions and livestock (pigs and cattle). (B) Daily activity patterns of domesticated pigs, cattle, and Yunnan snub-nosed monkeys, illustrating periods of potential temporal overlap. (C) Villages, farmland, and habitat of Yunnan snub-nosed monkeys in the study area. (D) Documented direct contact between Yunnan snub-nosed monkeys and pigs. (E) Documented direct contact between Yunnan snub-nosed monkeys and cattle.

Microorganisms can be transmitted between hosts through direct or indirect contact (shared environments) and lead to convergence of gut microbiome in the same environment.^40^^,^^41^^,^^42^ It has been shown that microbial transfer can occur between the environment and amphibian skin,^43^ and the seasonal environmental changes (water or soil) can lead to the amphibian commensal microbiome carrying a microbiome of putative corresponding environmental origin.^44^ In this study, we found higher similarity between the gut microbiome of cattle and Yunnan snub-nosed monkeys compared to pigs and Yunnan snub-nosed monkeys. We speculated that the shared environment may be the reason for the similarity in their gut microbiome (Figure 5). *Pandoraea* was found in both the gut microbiome of Yunnan snub-nosed monkeys and cattle. We found that the gut microbiome of cattle has a potential contribution to Yunnan snub-nosed monkeys, with *Pandoraea* accounting for 69.4%. In addition, the soil-derived microbiome may contribute to the cattle gut microbiome, of which 81.11% were classified as *Pandoraea*. Soil is considered to carry pathogens to can infect animals and humans.^45^ *Pandoraea* is predominantly isolated from patients with cystic fibrosis while also being widely distributed in natural environments (water and soil).^46^ We hypothesized that *Pandoraea* and other microorganisms may have been transmitted to cattle through the environment, and then transferred between animals through direct contact or shared environments, resulting in the existence of *Pandoraea* and a similar microbial distribution between cattle and Yunnan snub-nosed monkeys.

Diet also critically influences in the structuring of the gut microbial communities. Yunnan snub-nosed monkeys that forage in the wild for bamboo shoots and giant pandas have been found to have putative convergence in encoding cyanide detoxification microbiome and genes.^20^ Similarly, different developmental lineages of mammals (aardvarks, anteaters, and aardwolves) demonstrated gut microbial convergence driven by shared myrmecophagous diet.^47^ Furthermore, diet-driven adaptation can lead to functional microbiome convergence across mammalian hosts, including humans.^48^ Our work revealed shared habitats and partial dietary overlap between cattle and Yunnan snub-nosed monkeys, with both species harboring the plant polysaccharide-degrading bacterium *Rikenellaceae RC9* gut group in their microbiomes,^49^ suggesting that adaptation to similar diets may drive the similarity in the distribution of gut microbiome in the two species. In conclusion, we speculated that shared environment and partial dietary overlap may drive the higher similarity of gut microbiome between Yunnan snub-nosed monkeys and cattle, which also suggests that grazing may influence the gut microbiome of wildlife.

The investigation analyzed the gut microbiome composition in Yunnan snub-nosed monkeys and domestic livestock (pigs and cattle). The results showed that grazing may contribute to the similarity of gut microbiome between Yunnan snub-nosed monkeys and cattle, which is likely driven by shared environment and dietary overlap. Additionally, we found the potential microbial transmission between livestock and the environment, and the environmental contamination caused by grazing. These results demonstrate that livestock grazing alters wildlife gut microbiomes and their habitats, necessitating managed grazing zones in endangered species conservation initiatives. This may help prevent the potential spread of pathogenic microorganisms and ARGs through contact between domestic and wild animals. In addition, our study helped to understand the potential impacts of grazing activities on wildlife and the environment, and can optimize the grazing policy and balance the relationship between animal husbandry and biodiversity conservation.

### Limitations of the study

The current microbial traceability analysis was limited by the absence of longitudinal sampling (e.g., seasonal variations) and fine-scale spatial data (e.g., spatial overlap between livestock grazing zones and Yunnan snub-nosed monkeys' activity ranges). The study revealed that grazing may cause potential pathogen transmission. However, existing hypotheses were subject to limitations due to uncertainties in taxonomic methods based on 16S RNA sequencing and the strain-level variability in bacterial pathogenicity. Future studies will implement shotgun metagenomic sequencing for analyzing pathogenic microorganisms, profiling antibiotic resistance genes, and assessing microbial similarity at finer taxonomic levels. In addition, the study will collect samples from wild populations of Yunnan snub-nosed monkeys that have no contact with domestic livestock. Comparative analysis will help further elucidate whether observed microbiome similarities between Yunnan snub-nosed monkeys and livestock result from microbial transmission.

## Resource availability

### Lead contact

Requests for further information and resources should be directed to and will be fulfilled by the lead contact, Lifeng Zhu (zhulf2020@126.com).

### Materials availability

This study did not generate new, unique reagents.

### Data and code availability

• The data of Yunnan snub-nosed monkeys, pigs, and cattle have been deposited at NCBI: PRJNA1285119. The graphical abstract was created by Biorender.
• This article does not report original code.
• Any additional information required to reanalyze the data reported in this article is available from the lead contact upon request.

## Acknowledgments

This research was supported by the project of the 10.13039/501100001809National Natural Science Foundation of China (No. 32470509; 32070454).

## Author contributions

Conceptualization and methodology, D.L. and L.Z.; investigation and data curation, W.X., C.G., X. C., H.L., X.W., and F.W.; software and formal analysis, H.L., X.W., and F.W.; writing – original draft preparation, W.X., C.G., and X. C.; writing – review and editing, D.L. and L.Z.; All authors have read and agreed to the published version of the article.

## Declaration of interests

The authors declare no competing interests.

## STAR★Methods

### Key resources table

**Table:** 

REAGENT or RESOURCE	SOURCE	IDENTIFIER
**Biological samples**		

Yunnan snub-nosed monkeys, pigs, cattle and soil	This study	NCBI: PRJNA1285119

**Critical commercial assays**		

Fast DNA SPIN Kit for Feces	MPBIO	Cat#MP116570200
E. Z.N.A. Soil DNA Kit	Omega Bio-tek	D5625-02

**Deposited data**		

Sequencing data	This study	NCBI: PRJNA1285119

**Software and algorithms**		

Trimmomatic (v0.33)	http://www.usadellab.org/cms/?page=trimmomatic	http://www.usadellab.org/cms/?page=trimmomatic
Lima (v2.13.0)	https://github.com/PacificBiosciences/barcoding/	https://github.com/PacificBiosciences/barcoding/
cutadapt (v1.9.1)	https://github.com/marcelm/cutadapt	https://github.com/marcelm/cutadapt
UCHIME (v4.2)	http://drive5.com/usearch/manual/uchime_algo.html	http://drive5.com/usearch/manual/uchime_algo.html
DADA2 (v1.16)	https://benjjneb.github.io/dada2/index.html	https://benjjneb.github.io/dada2/index.html
UCLUST (v1.2.22q)	https://www.drive5.com/usearch/manual/uclust_algo.html	https://www.drive5.com/usearch/manual/uclust_algo.html
QIIME2 (v2022.2.0)	https://qiime2.org	https://qiime2.org
R (v4.1.0)	https://www.r-project.org/	https://www.r-project.org/

### Method details

#### Research site

The research was carried out in Xiangguqing (99°22′E, 27°37′N), located within the Baimaxueshan Nature Reserve at the Hengduan Mountains’ southern boundary, Yunnan Province, China(Figure 5). The region features a plateau monsoon climate with notable seasonal variation in temperature and rainfall.^50^ The study focused on evergreen broad-leaved forests, coniferous forests, and shrublands at elevations ranging from 2,600 to 3,200 meters. Local communities reside on the periphery of the reserve. Their livestock—primarily cattle and pigs—are free-ranging. Pigs forage daily in the mountainous areas of the reserve, ascending in the morning and returning to the village by dusk. Cattle exhibit wider daily movement ranges, dispersing over longer distances. To accommodate this, villagers practice open grazing, allowing cattle to roam freely within the mountainous terrain. Under these conditions, we quantified the diurnal activity patterns of domestic pigs, cattle, and Yunnan snub-nosed monkeys (*Rhinopithecus bieti*) and documented evidence of contact between these domestic species and the monkey populations. The monkey group involved in this study became semi-habituated and provisioned following a natural separation from the wild population in May 2008.^51^ From January 7, 2021, to April 9, 2022, the observed group comprised eight one-male units (OMUs) and one all-male unit (AMU), with a total population fluctuating between 59 and 68 individuals.

#### Sample collection

Based on consistent behavioral observations, Yunnan snub-nosed monkeys exhibit predictable defecation patterns following feeding. From 13:00 to 15:00 daily, we observed individuals from a distance of 20–30 meters and collected fresh fecal samples immediately after defecation. Between March 6 and April 26, 2022, fresh feces from cattle and pigs were collected within the study area. When collecting fecal samples, the superficial layer (approximately 1 cm thick) should be aseptically removed using sterile forceps. The inner fecal material was then carefully transferred into a sterile collection tube to minimize potential contamination from soil. The soil samples were collected from four different cardinal directions 1 meters away from the fecal samples, after discarding the top 1–2 cm surface layer using a sterile tool. All feces and soil samples were placed into sterile, pre-labeled tubes using aseptic techniques, stored in portable refrigeration units during fieldwork, and preserved at ultra-low temperature (-80°C) after return to the laboratory. In total, we obtained 165 samples, including 106 from Yunnan snub-nosed monkeys and 59 from livestock (30 pigs and 29 cattle). Sample metadata are detailed in Table S1.

#### DNA extraction and 16S rRNA sequencing

Genomic total DNA was extracted from fecal samples using the Fast DNA SPIN Kit for Feces (MPBIO, CA, USA),^52^ and the DNA from soil was extracted by the E. Z.N.A. Soil DNA Kit (Omega Bio-Tek, Norcross, GA, USA), following the manufacturer’s protocols. Concentration and integrity were assessed using a Qubit Fluorometer and 1% agarose gel electrophoresis. DNA was diluted to 1 ng/μL for PCR amplification of the whole 16S rRNA, using primer 27F(AGRGTTYGATYMTGGCTCAG) and 1492R(RGYTACCTTGTTACGACTT). PCR reactions were prepared with Phusion® High-Fidelity PCR Master Mix (New England Biolabs), 0.2 μM of each primer, and approximately 10 ng of template DNA. Thermocycling involved an initial denaturation at 98 °C (1 min), followed by 30 cycles at 98 °C (10 s), 50 °C (30 s), and 72 °C (60 s), and a final extension at 72 °C for 5 minutes. Amplicons were assessed on 2% agarose gels; those with clear bands (1400–1600 bp) were selected, pooled in equal ratios, and purified with the GeneJET Gel Extraction Kit (Thermo Scientific). Sequencing libraries were constructed using the NEBNext® Ultra™ DNA Library Prep Kit (NEB, USA), quality-checked on the Agilent Bioanalyzer 2100 and Qubit 2.0 Fluorometer, and sequenced using the Pacbio Sequel IIe platform.

#### Bioinformatic and statistical analyses

Raw sequencing data were subjected to quality control via Trimmomatic (v0.33),^53^ which filtered low-quality bases (Q < 20), implemented sliding window trimming (50 bp), and removed short reads (< 1200 bp). Subsequently, barcode and primer sequences were identified and trimmed using Lima (v2.13.0) and Cutadapt (v1.9.1). Finally, chimeric sequences were detected and removed with UCHIME (v4.2). The resulting high-quality sequences from each sample were then used for downstream analyses.

High-quality sequences were processed using DADA2 (v1.16)^54^ for denoising and error correction, generating an amplicon sequence variant (ASV) table at a 100% similarity threshold. Representative ASV sequences were taxonomically classified against the SILVA^55^ database using the UCLUST (v1.2.22q) classifier.^56^ To evaluate the adequacy of sequencing depth, the number of observed ASVs for each sample was calculated across a gradient of sequencing depths, using 200 sequences as the increment. To assess α diversity, the amplicon sequence variant (ASV) table was rarefied using the QIIME 2 (v2022.2.0) diversity alpha command (Figure S4). All samples were rarefied to a depth of 5,082 sequences to ensure even sampling effort for downstream alpha and beta diversity analyses. Phylogenetic Diversity (PD), Chao1, Shannon, and Simpson indices were subsequently calculated, and boxplots^57^ visualizing these indices were generated in R (v4.1.0) using the ggplot2 package. Community composition at phylum, family, and genus levels was profiled using relative abundance plots and compared statistically with the Kruskal–Wallis test, implemented via the kruskal.test() function in R (v4.1.0). We performed ANCOM-BC to identify taxa with significant differential abundance. We integrated Phylogenetic Cluster Analysis and Taxonomic Bar Plots for Microbial Community Composition.^58^ Corresponding visualizations were also produced using ggplot2, and the phylogenetic tree was constructed using the QIIME 2 (v2022.2.0) phylogeny module with the align-to-tree-mafft-fasttree pipeline.

Between-group differences (β-diversity) were analyzed using Bray-Curtis dissimilarities and visualized via hierarchical clustering and PCoA based on weighted UniFrac distances.^59^ The distance matrix was computed with the diversity beta module in QIIME 2 (v2022.2.0), and visualizations were generated using the ggplot2 package in R (v4.1.0). Venn diagrams^60^ were generated using the ggplot2 package in R (v4.1.0) to visualize the number of shared and unique amplicon sequence variants (ASVs). To infer potential microbial sources, we employed SourceTracker and indicator species analysis. The SourceTracker analysis used a Bayesian probability model, comparing the composition of “sink” communities against potential “source” communities to quantitatively estimate the contribution of each source to the sink microbiota.^61^ In the study, we applied SourceTracker to estimate the contributions of different microbiomes (from monkeys, livestock, and soil) to each host group. Indicator species can serve as bioindicators for specific habitats or host types. We performed the indicator species analysis using the indicspecies package in R (v4.1.0),^62^ and the resulting associations were visualized using Cytoscape.^63^

### Quantification and statistical analysis

Phylogenetic Diversity (PD), Chao1, Shannon, and Simpson indices were calculated using the R (v4.1.0). Significance between groups was assessed using the Wilcoxon rank-sum test (Figures 1A, and S1A–S1C). Differences in the relative abundance of taxa across the phylum, family and genus levels were evaluated using the Kruskal-Wallis test (Figures 1D, 1E, and S1E). Box plots showing gut microbial composition inter-group distances were analyzed using Student's t-test (Figure 2A). Group differences in community composition were visualized using Principal Coordinate Analysis (PCoA) and statistically tested with a Permutational Multivariate Analysis of Variance (PERMANOVA) based on weighted UniFrac distances with 999 permutations (Figure 2B). Differential abundance analysis of microbial taxa was performed using Analysis of Compositions of Microbiomes with Bias Correction (ANCOM-BC) (Figures S3A and S3B).

## Supplemental information

Document S1. Figures S1–S4

Table S1. The analysis of differential abundant phyla using ANCOM-BC

Table S2. The analysis of differential abundant families using ANCOM-BC

Table S3. The sample information in this study
