# Supplementary material for: Evidence for the effects on the wildlife gut microbiome by grazing: The potential gut microbiota transmission between Yunnan snub-nosed monkeys (Rhinopithecus bieti) and sympatric livestock
Source: iScience. 2025 Nov 19;28(12):114147. doi: 10.1016/j.isci.2025.114147 (PMC12719169; doi:10.1016/j.isci.2025.114147)
Supplement: Document S1. Figures S1–S4 [file mmc1.pdf]

iScience, Volume *28*

Supplemental information

Evidence for the effects on the wildlife gut microbiome by grazing: The potential gut microbiota transmission between Yunnan snub-nosed monkeys (*Rhinopithecus bieti*) and sympatric livestock

Wancai Xia, Chenyi Gao, Xinyuan Cui, Hong Li, Xueyu Wang, Fan Wang, Lifeng Zhu, and Dayong Li

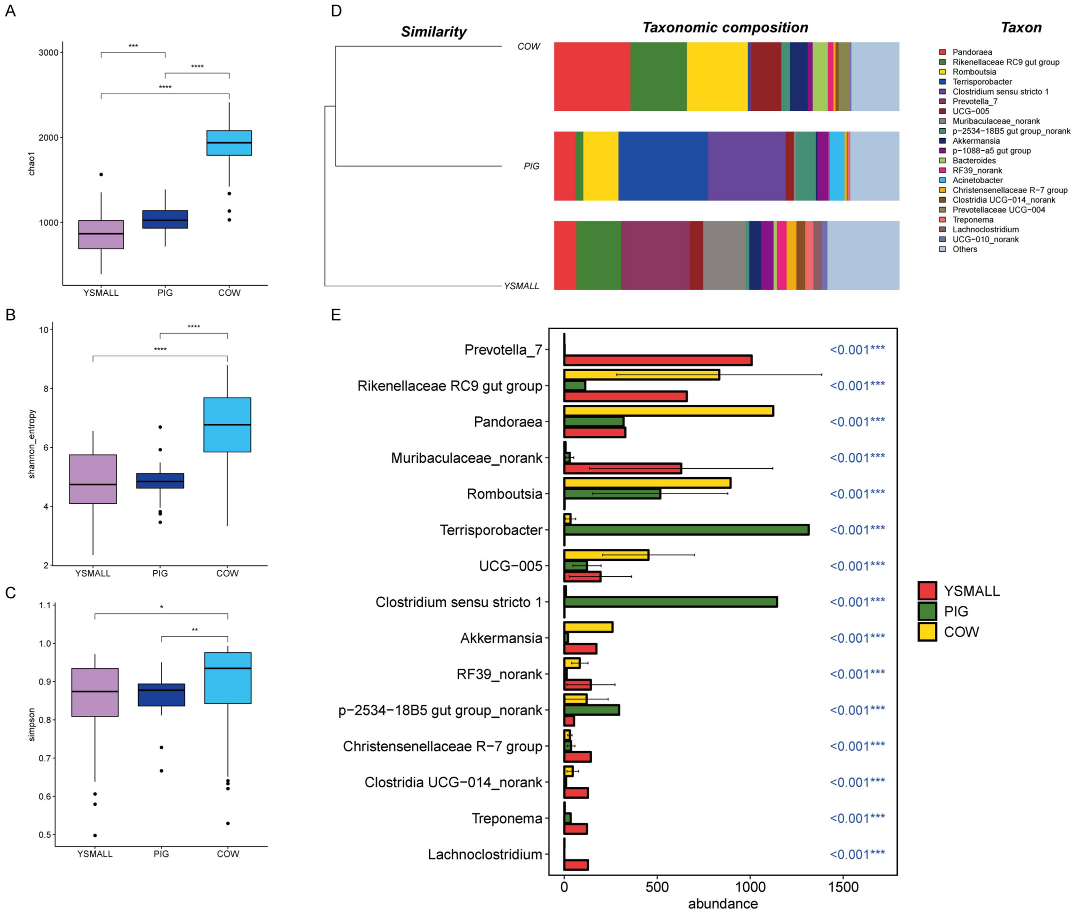

Figure S1. Gut microbial composition in Yunnan snub-nosed monkeys (YSMALL), pigs (PIG), and cattle (COW), related to Figure 1. n = 106 for YSMALL, n = 30 for PIG, n = 29 for COW. ***p < 0.001.

(A-C) Alpha diversity of the gut microbiome. Diversity was measured using the Chao1 (A), Shannon (B), and Simpson indices (C).

(D) Relative abundances of dominant genera in the gut microbiome of Yunnan snub-nosed monkeys, pigs, and cattle.

(E) Taxa with significant differences in the gut microbiome at the genus level in Yunnan snub-nosed monkeys, pigs, and cattle.

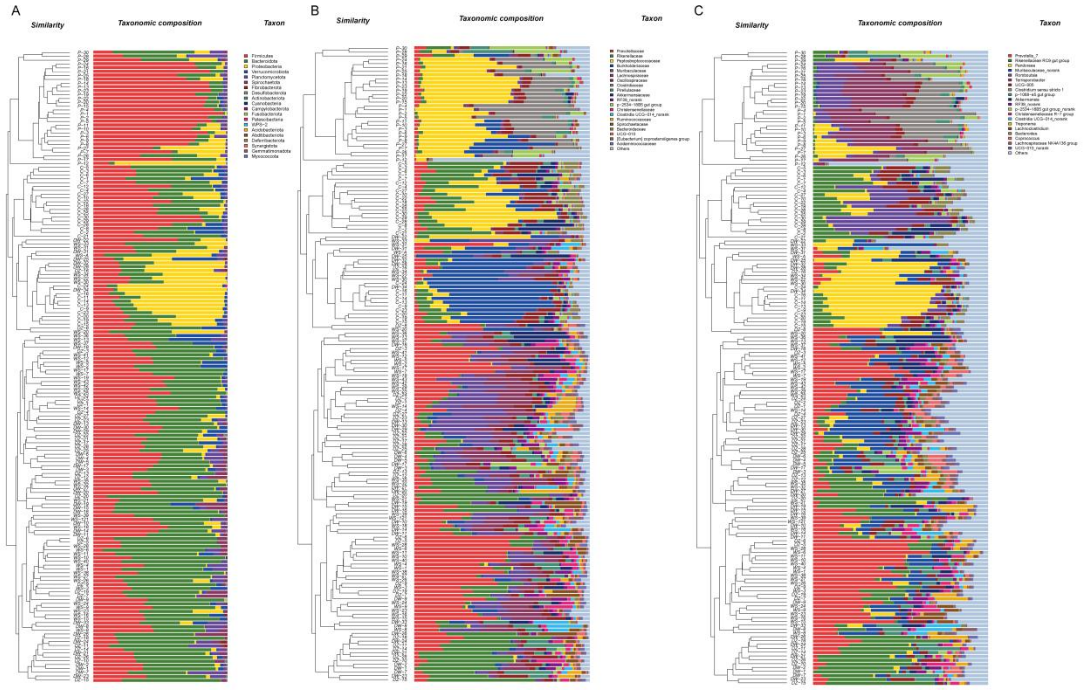

Figure S2. Taxonomic composition of the gut microbiome across samples at the phylum, family, and genus levels, related to Figure 1. n = 106 for YSMALL, n = 30 for PIG, n = 29 for COW.

(A) Phylum-level composition.

(B) Family-level composition.

(C) Genus-level composition.

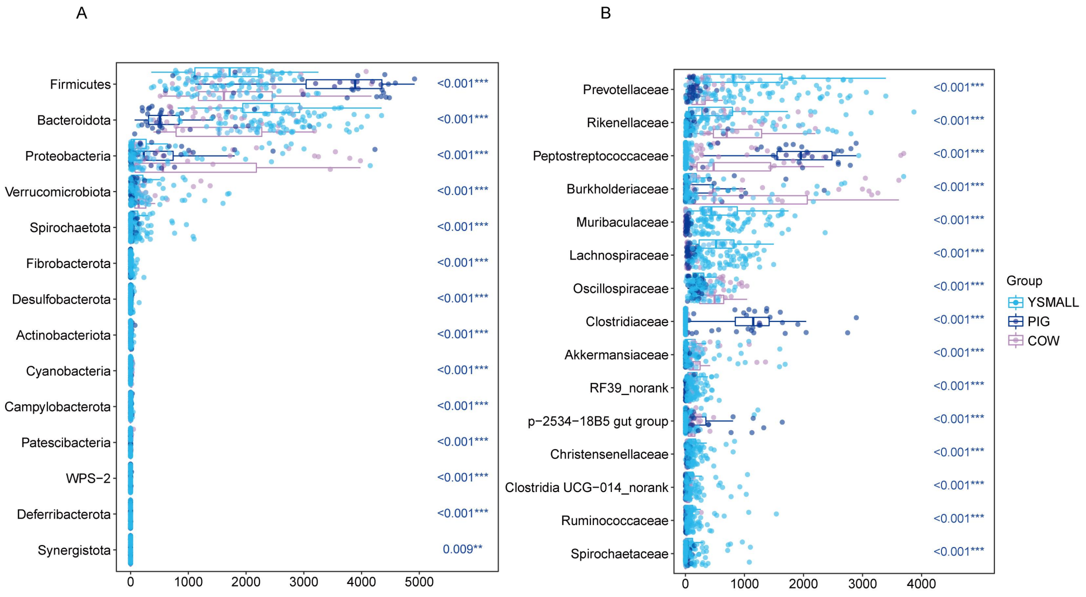

Figure S3. Significantly different taxa in the gut microbiome of Yunnan snub-nosed monkeys, pigs, and cattle at the (A) phylum and (B) family levels, related to Figure 1. Data are presented as medians with lower and upper quartiles; individual points represent different samples. **p < 0.01, ***p < 0.001.

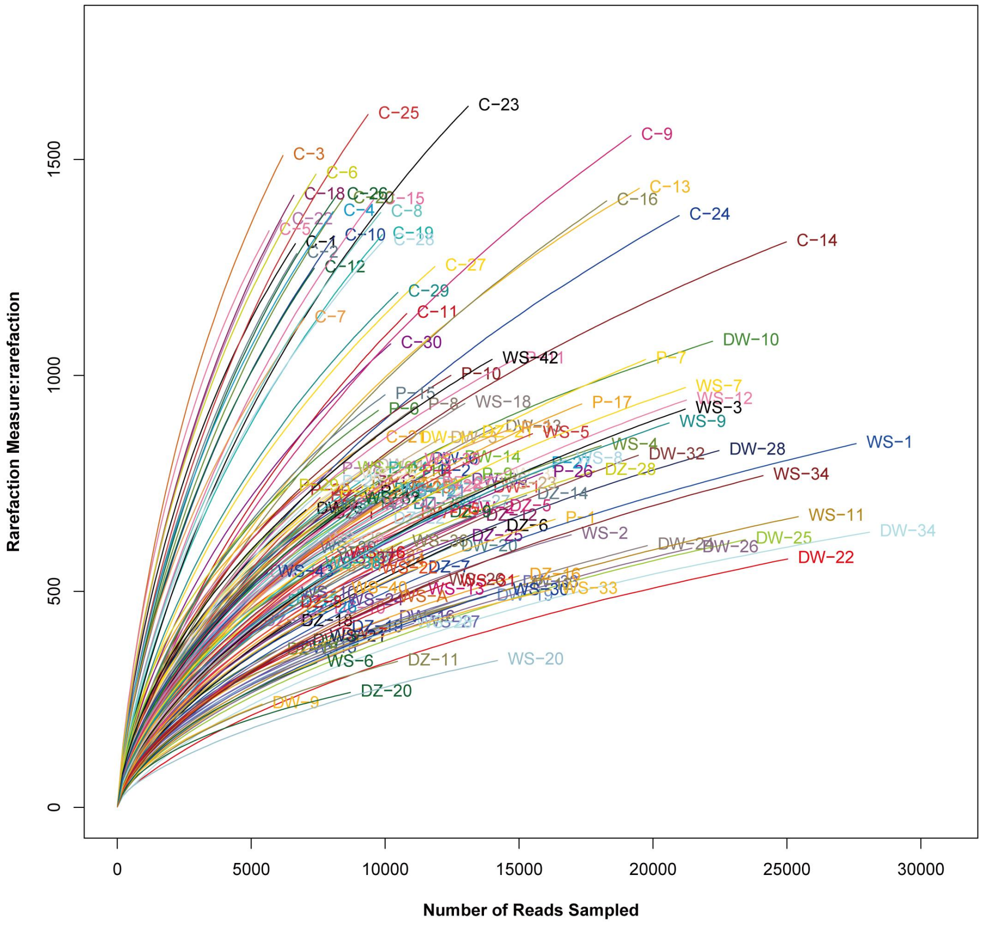

Figure S4. Rarefaction curves based on raw sequence data for all samples.
